# Editorial: Unveiling the neurobiological underpinnings of cognitive dysfunction in patients with schizophrenia

**DOI:** 10.3389/fpsyt.2024.1511643

**Published:** 2024-11-13

**Authors:** Tomiki Sumiyoshi, Luca De Peri, Stefano Barlati

**Affiliations:** ^1^ Department of Preventive Intervention for Psychiatric Disorders, National Center of Neurology and Psychiatry, Tokyo, Japan; ^2^ Department of Health and Social Care Cantonal Socio-Psychiatric Organization, Mendrisio, Switzerland; ^3^ Department of Clinical and Experimental Sciences, University of Brescia, Brescia, Italy

**Keywords:** schizophrenia, cognition, neurobiologic basis, intervention, phenomenology

Schizophrenia is a serious mental disorder that affects up to 1% of the population worldwide. Most patients with the illness suffer from disturbances of key aspects of cognitive function, e.g., neurocognition and social cognition, which worsen real-world functional outcomes. For example, neurocognitive impairment affects up to 75% of patients across multiple domains including processing speed, working memory, verbal memory, attention, executive functioning, while emotional processing, theory of mind, attributional bias, and social perception represent key domains of social cognition which are typically dysfunctional in schizophrenia. Several features of cognitive impairment are commonly found across schizophrenia and mood disorders, as well as their prodromal stages, indicating its transdiagnostic importance. Specifically, this *Topic* presents the cutting-edge knowledge on advanced assessments, neurobiological underpinnings, and interventional endeavors targeting cognitive impairment of schizophrenia.

Disturbances of neurocognition and social cognition of schizophrenia have been shown to be associated with both genetic and environmental factors. Among them, the genetic contribution is supported by Bae et al. who report poor performance on a test of facial emotion-recognition both in schizophrenia patients and their first-degree relatives. In this line, Deng et al. found an association between increased body mass index and neurocognition in first-episode drug-naive patients, also suggesting genetic susceptibility of cognitive impairment. On the other hand, the influence of socio-psychological (environmental) factors was reported by Peng et al. who observed an additive adverse effect of childhood trauma on cognition in patients with schizophrenia. Importantly, the association between cognitive symptoms and social function in individuals with at-risk mental states, as reported by Montemagni et al., underscore that cognitive impairment leads to poor functional outcome across stages of schizophrenia.

Neurobiological mechanisms of cognitive impairment of schizophrenia have been a Research Topic of intensive investigations based on data from a variety of research modalities. For example, Liang et al. employed resting-state functional magnetic resonance imaging and found increased levels of low-frequency fluctuation, an indicator of spontaneous and intrinsic neuronal activity, in childhood and adolescence-onset schizophrenia. Specifically, a relationship was noted between this imaging parameter and the performance on the tests of some cognitive domains. Another line of neurophysiological markers of cognitive function comes from magneto-electrical paradigms, e.g., electroencephalography and magnetoencephalography (MEG). By using MEG, Okazaki et al. reported altered auditory change-detection responses in patients with schizophrenia as a neural substrate for misattribution of inner experience to external agents. Based on the membrane phospholipid hypothesis, Higuchi et al. found associations between cognitive function and altered unsaturated fatty acid composition in the membrane of erythrocytes in antipsychotic-free schizophrenia patients.

Substantial efforts have been directed to the development of effective treatments of cognitive impairment of schizophrenia, with pharmacotherapy, non-invasive brain stimulation (NIBS) and cognitive rehabilitation as representative approaches. Accordingly, Li et al. presented data indicating domain-specific gender differences in cognitive improvements in patients with first-episode schizophrenia treated with antipsychotic drugs for two months. As a safe and feasible NIBS method, transcranial direct current stimulation was used by Yamada et al. who argued the advantage of stimulation of prefrontal cortical areas for enhancing neurocognition and higher levels of functional outcome, while disturbances of social cognition may be alleviated specifically when temporal brain regions are stimulated. A protocol of a randomized controlled study was introduced by Kubota et al., which aims to determine if combined treatment with cognitive remediation and a particular antipsychotic drug would improve neurocognitive function more effectively compared to the combination with another antipsychotic drug.

Overall, the papers contributed to this Research Topic provide a broad overview of the nature and underlying mechanisms of cognitive deficits of schizophrenia, as well as promising interventions to attain better outcomes for patients with this debilitating disorder.

